# Cerebral Perfusion Pressures and Implications on Clinical Outcomes and Medical Management in Cryptococcal Meningitis

**DOI:** 10.1093/ofid/ofaf451

**Published:** 2025-08-01

**Authors:** Mahsa Abassi, Cody Hou, Ann Fieberg, Biyue Dai, Abdu K Musubire, Jetter Robertson, Lillian Tugume, Kenneth Ssebambulidde, Edwin Nuwagira, Conrad Muzoora, Darlisha A Williams, David R Boulware, David B Meya, ASTRO study team, Edward Mpoza, Reuben Kiggundu, Katelyn A Pastick, Kenneth Ssebambulidde, Andrew Akampurira, Darlisha A Williams, Ananta S Bangdiwala, Abdu K Musubire, Melanie R Nicol, Cynthia Ahimbisibwe, Florence Kugonza, Carolyne Namuju, Kiiza K Tadeo, Paul Kirumira, Michael Okirwoth, Tonny Luggya, Julian Kaboggoza, Eva Laker, Stewart Walukaga, Emily E Evans, Anna Stadelman, Andrew G Flynn, Ayako W Fujita, Richard Kwizera, Sarah M Lofgren, Fiona V Cresswell, Bozena M Morawski

**Affiliations:** Division of Infectious Diseases, Department of Medicine, University of Minnesota, Minneapolis, Minnesota, USA; Division of Infectious Diseases, Department of Medicine, University of Minnesota, Minneapolis, Minnesota, USA; Division of Biostatistics and Health Data Science, School of Public Health, University of Minnesota, Minneapolis, Minnesota, USA; Division of Biostatistics and Health Data Science, School of Public Health, University of Minnesota, Minneapolis, Minnesota, USA; Infectious Disease Institute, College of Health Sciences, Makerere University, Kampala, Uganda; Department of Neurology, University of Minnesota, Minneapolis, Minnesota, USA; Infectious Disease Institute, College of Health Sciences, Makerere University, Kampala, Uganda; Infectious Disease Institute, College of Health Sciences, Makerere University, Kampala, Uganda; Department of Medicine, Mbarara University of Science and Technology, Mbarara, Uganda; Department of Medicine, Mbarara University of Science and Technology, Mbarara, Uganda; Division of Infectious Diseases, Department of Medicine, University of Minnesota, Minneapolis, Minnesota, USA; Division of Infectious Diseases, Department of Medicine, University of Minnesota, Minneapolis, Minnesota, USA; Infectious Disease Institute, College of Health Sciences, Makerere University, Kampala, Uganda

**Keywords:** cerebral perfusion pressure, cryptococcal meningitis, intracranial pressure, mean arterial pressure

## Abstract

**Background:**

In cryptococcal meningitis, increased intracranial pressure (ICP) is associated with worse outcomes and increased mortality. We sought to understand how changes in ICP and mean arterial pressure (MAP) affect cerebral perfusion pressure (CPP) and influence clinical outcomes.

**Methods:**

We performed a secondary data analysis of a prospective cohort of Ugandan adults with HIV-associated cryptococcal meningitis. We summarize demographic variables, clinical presentation, and 2-week survival by CPP and MAP groups.

**Results:**

Among 593 participants, 41% had low CPP <70 mm Hg, 54% had normal CPP 70–100 mm Hg, and 5% had high CPP >100 mm Hg. There was no association between baseline CPP and 2-week mortality. As a time-varying covariate, we observed a 39% increased risk of 2-week mortality with CPP levels <70 or >100 mm Hg (hazard ratio [HR] 1.39; 95% confidence interval [CI] 1.02–1.88, *P* = .04). Among 686 participants with baseline MAP measurements, there was an increased risk of 2-week mortality among people with low MAP <70 mm Hg (HR 1.80; 95% CI 1.01–3.20; *P* = .047) or high MAP >100 mm Hg (HR 1.47; 95% CI 1.08–1.99; *P* = .014) compared with normal MAP 70–100 mm Hg. We identified 4 clinical profiles based on MAP, CPP, and ICP measurements: (1) uncompensated intracranial hypertension (low CPP, elevated ICP, and low MAP), (2) compensated intracranial hypertension (normal CPP, elevated ICP, and MAP), (3) cerebral hypoperfusion (low CPP and low MAP), and (4) cerebral hyperperfusion (high CPP and high MAP).

**Conclusions:**

In cryptococcal meningitis, there is an intricate relationship between ICP, MAP, and CPP. We provide a concept framework using data from a clinical cohort and recommendations for clinical management.

## Teaser

Cerebral perfusion pressure emerges as a critical driver of outcomes in HIV-associated cryptococcal meningitis, reflecting the dynamic interplay between intracranial pressure and mean arterial pressure. Deviations from the optimal perfusion thresholds are associated with worse clinical outcomes, highlighting the value of individualized hemodynamic strategies.

Cryptococcal meningitis is the most common cause of meningitis among people living with HIV and is estimated to cause 19% of all AIDS-related mortality [1]. Aside from high mortality rates, cryptococcal meningitis survivors also suffer long-term disability, including persistent neurocognitive deficits, motor deficits, and vertigo [2, 3]. The mechanism for persistent neurocognitive deficits among cryptococcal meningitis survivors is not fully understood and may be secondary to cerebral ischemia. While the true extent of cerebral ischemia in cryptococcal meningitis is unknown, imaging studies suggest that up to one-third of persons have evidence of infarction, more commonly occurring in those HIV seronegative [4–7].

The pathophysiology underlying the development of cerebral ischemia in cryptococcal meningitis is not fully understood. Studies have suggested that in cryptococcosis, endarteritis of the subarachnoid vessels, as well as granulomatous inflammation and subsequent fibrosis of the subarachnoid space, can lead to small vessel occlusion and infarction [6, 8, 9]. Chang et al. [10] utilizing noninvasive cerebral hemodynamic monitoring, demonstrated evidence of noncritical cerebral artery stenosis and disrupted cerebral hemodynamics during cryptococcal meningitis. Additionally, the accumulation of cryptococcal yeasts in the arachnoid granulations obstructs the reabsorption of cerebrospinal fluid (CSF), leading to increased intracranial pressures (ICPs) [9, 11–13]. Increases in ICP may potentially lead to decreases in cerebral perfusion pressures (CPPs), resulting in cerebral hypoperfusion and subsequent ischemia [14, 15].

CPP, which represents the pressure gradient required to drive oxygen delivery to cerebral tissues, is the difference between mean arterial pressure (MAP) and ICP (CPP = MAP-ICP) [16, 17]. Under normal conditions, CPPs typically range from 70 to 100 mm Hg, and maintaining CPP within the physiological range is crucial for ensuring adequate cerebral blood flow and central nervous system tissue oxygenation [16, 17]. Derangements in either ICP or MAP can lead to either cerebral hypo- or hyperperfusion, both of which can have detrimental effects on cerebral blood flow and tissue oxygenation. We sought to understand how intracranial and/or MAP levels impact CPP in individuals with cryptococcal meningitis. Additionally, we aimed to understand how deviations from optimal CPP impacts clinical presentation and mortality in cryptococcal meningitis.

## METHODS

We conducted a prospective cohort study of Ugandan adults with HIV-associated cryptococcal meningitis enrolled into 2 clinical trials which tested adjunctive sertraline to the treatment of cryptococcal meningitis, from 2013 to 2017 [18, 19]. Participants were recruited and enrolled from 2 referral hospitals in Kampala and Mbarara, Uganda. Both trials were approved by the University of Minnesota and the Uganda institutional review boards. Written informed consent was obtained from participants or their surrogate. Participants received amphotericin B deoxycholate 0.7–1.0 mg/kg/day and oral fluconazole 800 mg/day for 14 days in addition to adjunctive sertraline at doses of 100–400 mg/day [19]. The addition of sertraline did not impact mortality or have an antifungal effect.

Daily hemodynamic monitoring during hospitalization included brachial blood pressure, heart rate, pulse oximetry, and temperature as part of routine care. ICPs were measured using bedside manometers during lumbar punctures at baseline, days 3, 7, 10, and 14, as well as any additional days at the discretion of the study physician for the management of raised ICPs. CPP was calculated as the difference between the MAP and ICP (CPP_mm Hg_ = MAP_mm Hg_ − [ICP_mm Hg_O__ × 0.0736 mm Hg/mm H_2_O]). The MAP was calculated as the average blood pressure during a single cardiac cycle (MAP = 1/3[SBP_mm Hg_] + 2/3[DBP_mm Hg_]). In this analysis, we used measurements of ICP and MAP taken on the same day at baseline.

Demographic variables, clinical presentation, and CSF characteristics at baseline were summarized by CPP tertiles (low CPP <70 mm Hg, normal CPP 70–100 mm Hg, and high CPP >100 mm Hg) and MAP tertiles (low MAP <70 mm Hg, normal MAP 70–100 mm Hg, and high MAP >100 mm Hg). We compared clinical outcomes, including the percentage of incident seizures [20] and new onset or sustained (present at baseline) altered mental status at days 7 and 14 by both CPP and MAP groupings. We tested for differences across groups using the χ^2^ test for categorical variables and the Kruskal–Wallis test for continuous variables.

We examined whether there was an association between 2-week mortality and either baseline CPP or MAP using Cox proportional hazards models. We additionally conducted a Cox proportional hazards analysis to assess the association between 2-week mortality and time-varying covariates for CPP (<70 or >100 mm Hg) and MAP (<70 or >100 mm Hg). All models were adjusted for the following variables measured at baseline and defined a priori: Glasgow Coma Scale (GCS) score <15 versus 15, CSF cryptococcal quantitative culture, and seizures [20–22]. A 2-sided type I error of 0.05 was used. We performed all analyses with SAS version 9.4 (SAS Institute, Cary, North Carolina).

## RESULTS

**Figure 1.**
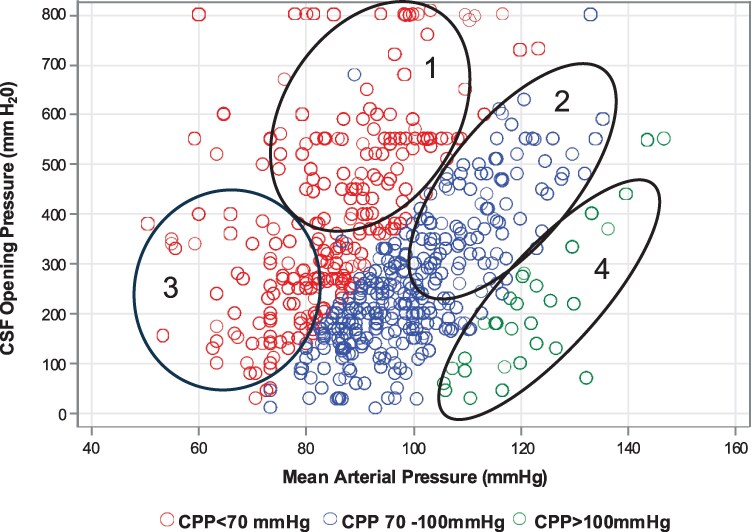
Scatterplot by systolic blood pressure, CSF opening pressure, and CPP. Scatterplot of individual measurements by systolic blood pressure, CSF opening pressure, and CPP. 1 = Uncompensated intracranial hypertension: low CPP, low MAP, high ICP. 2 = Compensated intracranial hypertension: normal CPP, high MAP, high ICP. 3 = Cerebral hypoperfusion: low CPP, low MAP. 4: Cerebral hyperperfusion; high CPP, high MAP.

From 2013 through 2017, a total of 695 participants with HIV and a positive CSF cryptococcal antigen (CrAg) by lateral flow assay were enrolled into 2 clinical trials. Approximately 99% (686/695) had baseline blood pressure measurements recorded and are included in the MAP analysis. Of the 686, 593 participants (85%) additionally had CSF opening pressure recorded and were included in the CPP analyses. At baseline, 41% (242/593) had low CPP <70 mm Hg (median 59 mm Hg; interquartile range [IQR] 51–65 mm Hg), 54% (321/593) had normal CPP 70–100 mm Hg (median 80 mm Hg; IQR 75–87 mm Hg), and 5% (30/593) had high CPP >100 mm Hg (median 105 mm Hg; IQR 102–109 mm Hg; Table 1). When grouping by baseline MAP, 15% (36/686) had low MAP <70 mm Hg (median 63 mm Hg; IQR 59–67 mm Hg), 62% (428/686) had normal MAP 70–100 mm Hg (median 88 mm Hg; IQR 82–94 mm Hg), and 32% (222/686) had high MAP >100 mm Hg (median 109 mm Hg; IQR 103–117 mm Hg; Supplementary Table 1). In persons with cryptococcal meningitis, when grouping by either CPP or MAP, 4 clinically distinct groupings emerged among people whose MAP or CPP was outside of the normal range: (1) uncompensated intracranial hypertension, (2) compensated intracranial hypertension, (3) cerebral hypoperfusion, and (4) cerebral hyperperfusion (Figure 1).

**Table 1.** Baseline Demographics, Clinical Presentation, and CSF Characteristics by CPP

	Baseline CPP						
	CPP <70 mm Hg Median (IQR) or n (%)		CPP 70–100 mm Hg Median (IQR) or n (%)		CPP >100 mm Hg Median (IQR) or n (%)		*P*-value*
Number of participants	N	242	N	321	N	30	
Demographics							
Age, y	242	33 (29–40)	321	36 (30–42)	30	38 (30–45)	<.01
Female	242	102 (42%)	321	121 (38%)	30	10 (33%)	.45
Clinical characteristics							
Weight, kg	195	52 (48–60)	271	53 (48–60)	24	54 (50–60)	.52
Receiving HIV therapy	242	135 (56%)	321	163 (51%)	30	15 (50%)	.44
CD4^+^ count cells/μL	228	15 (6–43)	308	17 (7–49)	28	11 (5–54)	.50
Creatinine, mg/dL	216	0.7 (0.5–0.9)	296	0.7 (0.6–0.9)	27	0.8 (0.6–1.1)	.06
Hemoglobin, g/dL	217	11.8 (10.3–13.2)	290	11.5 (9.9–12.9)	28	12.8 (9.2–14.7)	.25
Systolic BP, mm Hg	242	110 (100–122)	321	126 (118–137)	30	152 (132–165)	-
Diastolic BP, mm Hg	242	70 (60–80)	321	85 (78–94)	30	106 (99–116)	-
Mean arterial pressure, mm Hg	242	84 (76–93)	321	99 (90–107)	30	120 (115–130)	-
Seizures	242	38 (16%)	321	41 (13%)	30	10 (33%)	<.01
GCS score <15	242	109 (45%)	321	121 (38%)	30	14 (48%)	.18
CSF characteristics							
Intracranial opening pressure, mm H_2_O	242	370 (260–550)	321	230 (168–330)	30	180 (92–274)	-
Opening pressure >250 mm H_2_O	185	(77%)	137	(43%)	9	(30%)	-
Total white cells, cells/μL	231	<5 (<5–40)	308	<5 (<5–45)	27	<5 (<5–80)	.65
CPP, mm Hg	242	59 (51–65)	321	80 (75–87)	30	105 (102–109)	-
Quantitative culture, log10 CFU/mL	237	4.7 (3.3–5.6)	316	4.5 (2.7–5.3)	29	4.6 (3.0–5.4)	.11
Protein, mg/dL	204	59 (24–99)	284	46 (22–100)	27	61 (33–129)	.23
Glucose, mg/dL	79	57 (36–95)	85	52 (37–87)	6	100 (31–121)	.64

Values are represented as percentages or median and interquartile range. CPP is calculated as the MAP minus (intracranial opening pressure × 0.073556127270818), where MAP = (1/3) × systolic BP + (2/3) × diastolic BP.

Abbreviations: BP, blood pressure; CFU, colony-forming units; CPP, cerebral perfusion pressure; CSF, cerebral spinal fluid; GCS, Glasgow Coma Scale; IQR, interquartile range.

^*^ *P*-values are compared across the 3 groups using the χ^2^ test for proportions or the Kruskal–Wallis test for continuous variables.

The first group consisted of individuals with low CPP, resulting from elevated ICP and low MAP, aligning with the pathophysiology of uncompensated intracranial hypertension (Figure 1). At baseline, individuals with low CPP (<70 mm Hg) had elevated intracranial opening pressures (median ICP 370 mm H_2_O; IQR 260–550 mm H_2_O) and lower MAPs (median MAP 84 mm Hg; IQR 76–93 mm Hg) compared with those with normal CPP or high CPP (Table 1). There were no significant differences in CSF quantitative cryptococcal cultures, CSF white blood cell count, CSF glucose, or CSF protein across CPP groups.

The second group was comprised of individuals with CPP falling within the physiological range, resulting from elevated MAPs and high intracranial opening pressure, aligning with the pathophysiology of compensated intracranial hypertension (Figure 1). Individuals with CPP 70–100 mm Hg had both elevated ICPs (median ICP 230 mm H_2_O; IQR 168–330 mm H_2_O) and elevated MAPs (median MAP 99 mm Hg; IQR 90–107 mm Hg; Table 1). Similarly, those with a high MAP (>100 mm Hg) had elevated ICPs (median ICP 332 mm H_2_O; IQR 212–480 mm H_2_O) and CPPs that were within the acceptable physiological range (median CPP 86 mm Hg; IQR 78–94 mm Hg; Supplementary Table 1).

The third group was comprised of individuals with low CPP resulting from low MAP, aligning with the pathophysiology of cerebral hypoperfusion (Figure 1). Participants with low MAP (<70 mm Hg) had lower CPPs (median CPP 41 mm Hg; IQR 29–53 mm Hg) when compared with CPPs when MAPs were either normal or high (Supplementary Table 1). Individuals with a low MAP at baseline were more likely to have a GCS score <15 (*P* = .007) compared with those with normal or high MAP.

The fourth group was comprised of individuals with high CPP, resulting from high MAPs without any significant elevations in ICPs, aligning with the pathophysiology of cerebral hyperperfusion (Figure 1). At baseline, individuals with high CPP (>100 mm Hg) had higher MAPs (median MAP 120 mmHg; IQR 115−130 mm Hg) and lower intracranial opening pressures (median ICP 180 mm H_2_O; IQR 92–274 mm H_2_O) compared with those with low or normal CPP (Table 1). Participants with high CPP were more likely to present with self-reported seizures (*P* < .01) and experience incident seizures during the first 7 days than those with normal or low CPP groups (incident seizures, low CPP: 9% [22/242]; normal CPP: 6% [18/321]; high CPP 20% [6/30]; *P* = .01; Table 2).

**Table 2.** Clinical Outcomes and 2-Week Mortality by Baseline CPP

	Clinical Outcomes by Baseline CPP Categories			
	CPP <70 mm Hg	CPP 70–100 mm Hg	CPP >100 mm Hg	
Number of participants	242	321	30	*P*-value*
Incident seizures (first 7 d), N^a^	242	321	30	
n (%)	22 (9%)	18 (6%)	6 (20%)	.01
Day 7 GCS score <15, N^a^	172	235	21	
n (%)	35 (20%)	44 (19%)	8 (38%)	.11
Day 14 GCS score <15, N^a^	133	147	16	
n (%)	27 (20%)	19 (13%)	3 (19%)	.25

	2-Week Mortality by Baseline CPP Categories				
	CPP <70 mm Hg		CPP 70–100 mm Hg	CPP >100 mm Hg	
Number of participants	242	*P*-value*	321	30	*P*-value**
Number of deaths (%)	73 (30%)		83 (26%)	10 (33%)	
Unadjusted HR (95% CI)	1.22 (0.89–1.68)	0.21	REF	1.33 (0.69–2.57)	.39
Adjusted HR (95% CI)^b^	1.14 (0.83–1.57)	0.41	REF	1.04 (0.52–2.11)	.91

Abbreviations: CI, confidence interval; CPP, cerebral perfusion pressure; GCS, Glasgow Coma Scale; HR, hazard ratio.

^a^n with data.

^b^Models were adjusted for baseline variables of GCS < 15, quantitative culture results, and seizures (yes/no).

^*^ *P*-values compare across the 3 groups using the χ^2^ test.

^**^ *P*-values for 2-week mortality are from Cox regression models.

**Figure 2.**
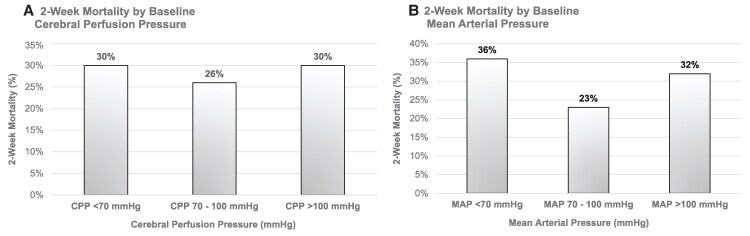
Two-week mortality by baseline (*A*) CPP and (*B*) MAP.

We assessed whether baseline CPP was associated with 2-week mortality. Death rates were higher in individuals who had either a baseline low CPP (30%) or high CPP (33%) when compared with those who had a normal baseline CPP (26%; Table 2, Figure 2, Supplementary Figure 1). While there was no association between baseline CPP and 2-week mortality in our unadjusted analysis, when assessing CPP as a time-varying covariate, we observed a 39% increased risk of 2-week mortality associated with either CPP levels < 70 mm Hg or >100 mm Hg (hazard ratio [HR] 1.39; 95% confidence interval [CI] 1.02–1.88, *P* = .04; Supplementary Table 3). We also assessed whether baseline MAP was associated with 2-week mortality. We found that low MAP (<70 mm Hg) was associated with an 80% increased risk in 2-week mortality (HR 1.80; 95% CI 1.01–3.20; *P* = .047) and high MAP was associated with a 47% increased risk of 2-week mortality (HR 1.47; 95% CI 1.08–1.99; *P* = .014) when compared with those with normal MAP (70–100 mm Hg; Figure 2, Supplementary Table 2). When adjusting for baseline GCS < 15, quantitative cryptococcal culture, and seizures, CPP and MAP were no longer associated with 2-week mortality.

## DISCUSSION

**Figure 3.**
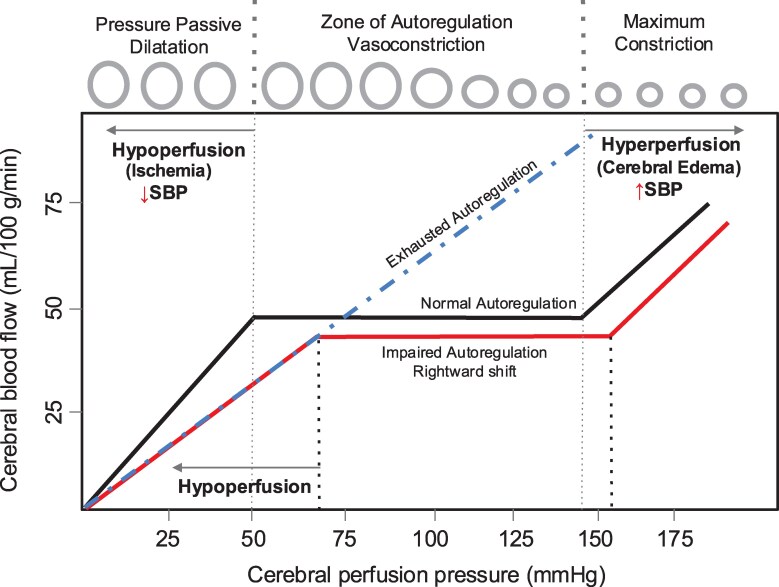
Proposed framework for cerebral autoregulation in cryptococcal meningitis. Figure adapted and modified from Toth et al [16]. Proposed model illustrating changes in cerebral autoregulation in the setting of cryptococcal meningitis. When autoregulatory function is intact (normal autoregulation), despite changes in CPP, cerebral blood flow is maintained at a constant level. When cerebral autoregulation is impaired, we postulate that the curve is rightward shifted (impaired autoregulation) such that higher perfusion pressures are needed to maintain cerebral blood flow. When perfusion pressure decreases below a new set critical point, cerebral hypoperfusion and ischemia ensue. When cerebral autoregulation is completely exhausted (blue line), cerebral blood flow passively changes in response to changing CPP. Elevated CPPs, leading to increased intravascular pressure, may induce fluid to leak from blood vessels into surrounding brain tissue, contributing to cerebral edema.

In this study, we highlight the importance of CPP as a key consideration in the management of cryptococcal meningitis. We identified 4 clinically relevant hemodynamic profiles based on CPP and MAP. CPP is commonly used as a surrogate measure of cerebral blood flow [17]. Under normal circumstances, cerebral autoregulation alters vascular resistance, by vasodilation or vasoconstriction of cerebral blood vessels, in response to changing CPPs, thereby maintaining adequate cerebral blood flow [17]. Based on our observations of MAP, ICP, and CPP, we propose a model of cerebral autoregulatory mechanisms in cryptococcal meningitis (Figure 3) [16]. In cases of cryptococcal meningitis, when cerebral autoregulation is preserved, despite changes in CPP in response to MAP and ICP, cerebral blood flow is maintained at near a constant level [17]. However, in cases of severe disease, cerebral autoregulation may be impaired, resulting in a rightward shift in the autoregulatory curve such that higher CPPs are needed to maintain cerebral blood flow and prevent cerebral hypoperfusion [23]. When cerebral autoregulatory mechanisms become exhausted, cerebral blood flow becomes dependent on blood pressure, such that cerebral blood flow comes to closely correlate with CPP [16].

**Figure 4.**
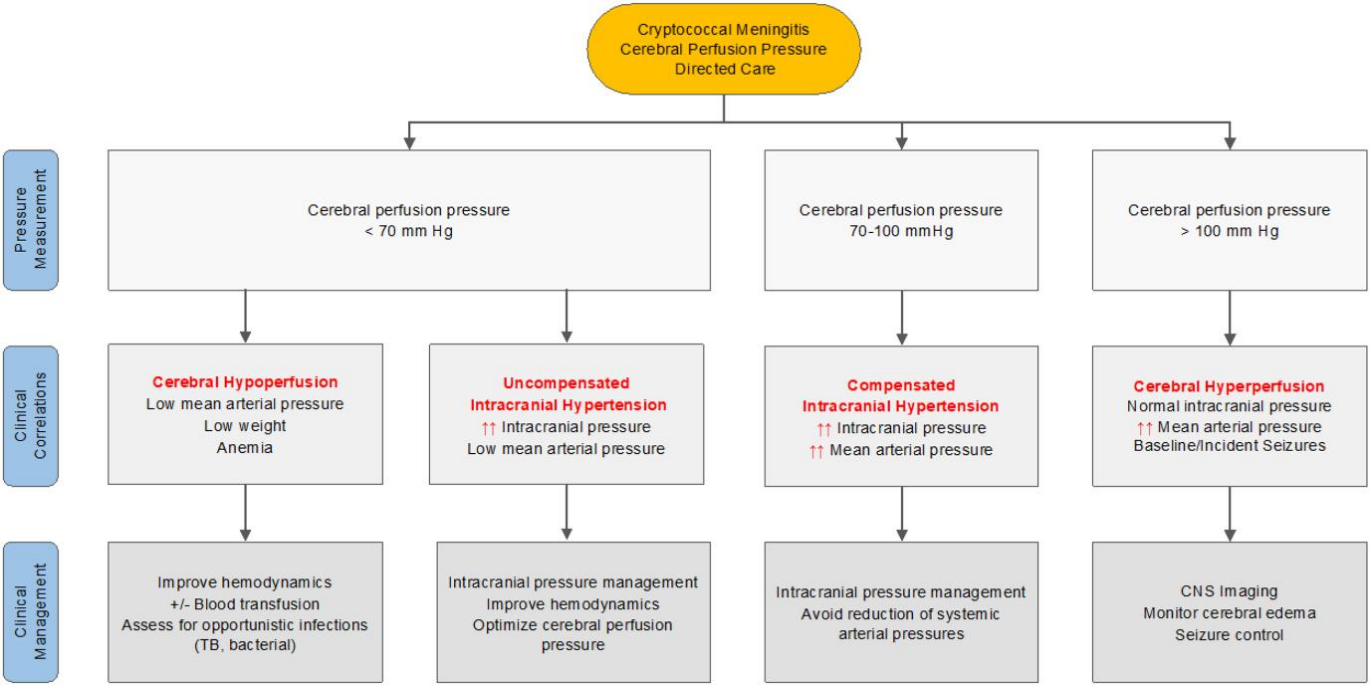
Clinical management of cryptococcal meningitis by baseline CPP. The proposed management algorithm is based on an initial CPP (CPP = MAP – ICP) measurement and corresponding clinical correlations.

In cases of acute brain injuries, monitoring ICP has become crucial to effective clinical management [24]. ICP monitoring serves not only to manage raised ICP but also serves as a method to quantify CPP through its relationship with MAP (CPP = MAP − ICP). CPP monitoring has been incorporated into traumatic brain injury guidelines as a bundle of care, in addition to monitoring MAP and ICP, with the aim of preventing secondary ischemic insults to the brain and reducing 2-week mortality postinjury [24]. Targeting CPP values of 60–70 mm Hg in traumatic brain injury has been shown to improve outcomes [25, 26]. Implementing an ICP − CPP (ICP/CPP)-oriented protocol in the management of cryptococcal meningitis may improve outcomes by optimizing CPPs to mitigate secondary insults of hypoperfusion on brain tissue. In fact, our findings would suggest that monitoring CPP may aid in distinguishing between 4 different physiological presentations, thereby informing a more nuanced approach to cryptococcal meningitis management (Figure 4).

In cases of uncompensated and compensated intracranial hypertension, enhancing CPP through the reduction of ICP should be the primary therapeutic strategy with the objective of averting potentially life-threatening cerebral hypoperfusion and subsequent neurological toxicities (ie, ischemia and seizures) [15]. Furthermore, optimization of MAPs in the setting of raised ICPs should also be an important consideration in cryptococcal meningitis management. Given that cerebral autoregulatory mechanisms may be impaired such that a higher CPP is required to maintain cerebral blood flow, allowing for elevated MAPs may allow for sustained cerebral blood flow in the setting of intracranial hypertension. Iatrogenic efforts to reduce systemic arterial pressures, while ICPs remain elevated, could have the detrimental effect of reducing CPP below a critical point leading to global brain ischemia.

We observed that low MAPs were associated with lower CPPs. In cases of cerebral hypoperfusion, low MAP was more frequently identified in individuals with a lower body weight and hemoglobin level, potentially indicating more advanced HIV disease at the time of presentation. Individuals with advanced HIV disease tend to be more vulnerable to co-infections with tuberculosis and other opportunistic infections, develop bacteremia, nosocomial infections, and generally have higher in-hospital mortality rates [27, 28]. Baseline anemia is also known to be strongly associated with low regional cerebral tissue oxygenation and an independent predictor of mortality in cryptococcal meningitis [11, 29]. In cases of sepsis-associated encephalopathy, impairment of cerebral autoregulation has been shown to be an independent predictor of worse outcomes [25]. Therefore, optimization of cerebral hemodynamics will necessitate a more aggressive approach to diagnosing and managing concurrent opportunistic and nosocomial infections as well as improving oxygen-carrying capacity thereby preventing cerebral hypoperfusion [28, 30].

We also observed that high CPP occurred when systemic arterial pressure was elevated without significant increases in ICP, leading to a state of cerebral hyperperfusion [23]. Individuals with elevated CPP, driven by increased MAP, presented more frequently with a higher percentage of self-reported seizures at baseline and incident seizures within the initial 7 days of cryptococcal meningitis therapy. In cases of traumatic brain injury, elevated CPPs stem from heightened cerebral capillary permeability to water and dilation of cerebral blood vessels, resulting in cerebral edema [31]. As arterial blood pressure rises, there is an accompanying increase in cerebral blood flow, CPP, and cerebral edema [31]. Worsening cerebral edema, leading to compression of cerebral tissues, might provide an explanation for the higher percentage of both baseline and new-onset seizures within the first 7 days in individuals with high CPPs. Identification of cerebral edema via imaging or clinical suspicion based on decreasing GCS score may provide an opportunity to detect seizure activity and incorporate antiepileptic therapy. In cases of worsening neurological dysfunction resulting from increasing cerebral edema, dexamethasone, a potent glucocorticoid, is used to reduce vascular permeability, limiting the entry of inflammatory cells and fluids, thereby reducing cerebral edema [31]. However, in cryptococcal meningitis, the use of dexamethasone is not recommended, as a randomized clinical trial demonstrated no mortality benefit and more adverse events with its use [32]. The possibility of incorporating an ICP/CPP protocol would permit a more targeted approach to the use of dexamethasone in the management of cryptococcal meningitis. Lastly, attempts to reduce MAPs should be done with caution, as cerebral autoregulatory mechanisms may be exhausted such that cerebral blood flow becomes dependent on arterial blood pressure. Decreasing blood pressure may have the detrimental effect of decreasing cerebral blood flow below a critical point, thereby precipitating cerebral hypoxia.

While we did not find a significant difference in 2-week mortality across CPP groups, we did observe a bimodal distribution in mortality. Individuals with either low (<70 mm Hg) or high (>100 mm Hg) CPPs at baseline had higher 2-week mortality than those with CPPs between 70 and 100 mm Hg. Since cerebral perfusion is a dynamic process, relying solely on a 1-time baseline measurement may oversimplify risk assessment throughout hospitalization for 2-week mortality. When we considered CPP as a time-varying covariate, we found that subsequent occurrences of either high or low pressures increased the risk of death by 39%. In a study involving individuals who suffered head injuries, a comparable trend in mortality was noted, with higher overall mortality rates observed when CPP fell below 55 mm Hg or exceeded 95 mm Hg [33]. Among individuals who suffered a head injury, those who did not survive had higher ICPs and lower CPPs compared with survivors [33]. Additionally, individuals with CPPs surpassing 95 mm Hg experienced higher rates of severe disability and adverse outcomes, including mortality and persistent vegetative states [33].

Our study has several limitations. Blood pressure and ICP measurements were not routinely performed simultaneously, and therefore, we are not able to account for any possible changes in MAPs prior to ICP measurements. While we have previously reported on regional tissue oxygenation (rSO_2_) using noninvasive monitoring by cerebral oximetry, we did not have cerebral oximetry readings in this study to correlate regional cerebral oxygen saturation with CPPs. A future study using cerebral oximetry or transcranial Doppler could allow the calculation of cerebral vascular resistance to determine disruptions in cerebral autoregulatory mechanisms in cryptococcal meningitis.

In conclusion, integrating neurological care and support into the treatment of cryptococcal meningitis is crucial. The calculation of CPP, through the measurement of ICP and MAP, is instrumental in identifying individuals at high risk for cerebral hypo- and hyperperfusion. Early intervention to address increased ICP and systemic hypotension is essential to maintain CPP within a physiologically acceptable range, thereby preventing secondary ischemic insults to the brain. High systemic arterial pressures, although justified in the presence of increased ICP, may be detrimental in persons with normal ICP. Further research is imperative to validate the efficacy of an ICP/CPP-oriented protocol in the management of cryptococcal meningitis.

## Supplementary Material

ofaf451_Supplementary_Data

## Supplementary Data

Supplementary materials are available at *Open Forum Infectious Diseases* online. Consisting of data provided by the authors to benefit the reader, the posted materials are not copyedited and are the sole responsibility of the authors, so questions or comments should be addressed to the corresponding author.
