# Supplementary material for: Cerebral Perfusion Pressures and Implications on Clinical Outcomes and Medical Management in Cryptococcal Meningitis
Source: Open Forum Infect Dis. 2025 Aug 1;12(8):ofaf451. doi: 10.1093/ofid/ofaf451 (PMC12359033; doi:10.1093/ofid/ofaf451)
Supplement: ofaf451_Supplementary_Data [file ofaf451_supplementary_data.docx]

| **Supplemental Table 1: Baseline Demographics, Clinical Presentation, and CSF Characteristics by Mean Arterial Pressure** | | | | | | | |
| --- | --- | --- | --- | --- | --- | --- | --- |
|  | **Baseline Mean Arterial Pressure (MAP)** | | | | | |  |
|  |  | **MAP <70 mmHg**  Median (IQR) or  N (%) |  | **MAP 70 to 100 mmHg**  Median (IQR) or  N (%) |  | **MAP >100 mmHg**  Median (IQR) or  N (%) | **p-value*** |
| **Number of Participants** | **N** | **36** | **N** | **428** | **N** | **222** |  |
| **Demographics** |  |  |  |  |  |  |  |
| Age, years | 36 | 38 (32 - 42) | 428 | 34 (29 - 40) | 222 | 36 (30 - 43) | <0.001 |
| Female | 36 | 15 (42%) | 428 | 180 (42%) | 222 | 78 (35%) | 0.23 |
| **Clinical Characteristics** |  |  |  |  |  |  |  |
| Weight, kg | 28 | 52 (48 - 60) | 369 | 52 (47 - 60) | 172 | 55 (50 - 60) | **0.03** |
| Receiving HIV therapy | 36 | 24 (67%) | 428 | 228 (53%) | 222 | 112 (51%) | 0.20 |
| CD4+ count, cells/μL | 34 | 32 (9 – 54) | 409 | 16 (7 - 48) | 208 | 15 (6 - 47) | 0.38 |
| Creatinine, mg/dL | 34 | 0.7 (0.5 – 1.0) | 391 | 0.7 (0.6 - 0.9) | 200 | 0.7 (0.6 - 0.9) | 0.90 |
| Hemoglobin, g/dL | 32 | 10.8 (9.9 - 12.7) | 391 | 11.3 (9.8 - 12.8) | 195 | 12.1 (10.3 - 13.6) | **<0.01** |
| Systolic BP, mmHg | 36 | 90 (82 - 99) | 428 | 116 (108 - 122) | 222 | 139 (131 - 147) | - |
| Diastolic BP, mmHg | 36 | 50 (41 – 50) | 428 | 74 (69 - 80) | 222 | 96 (90 – 101) | - |
| Mean arterial pressure, mmHg | 36 | 63 (59 - 67) | 428 | 88 (82 - 94) | 222 | 109 (103 - 117) | - |
| Seizures | 36 | 7 (19%) | 428 | 48 (11%) | 222 | 44 (20%) | **<0.01** |
| Glasgow Coma Scale score <15 | 36 | 24 (67%) | 428 | 162 (38%) | 222 | 104 (47%) | **<0.001** |
| **CSF Characteristics** |  |  |  |  |  |  |  |
| Intracranial opening pressure, mmH2O | 26 | 305 (155 - 400) | 371 | 250 (170 -370) | 198 | 332 (212 - 480) | **<0.001** |
| Opening pressure >250 mmH2O | 15 | (58%) | 183 | (49%) | 133 | (67%) | **<0.001** |
| Total white cells, cells/μL | 31 | <5 (<5 - 40) | 394 | <5 (<5 - 40) | 209 | <5 (<5 - 55) | 0.88 |
| Cerebral perfusion pressure, mmHg | 26 | 41 (29 - 53) | 370 | 70 (60 - 76) | 197 | 86 (78 - 94) | **-** |
| Quantitative culture, log10 CFU/mL | 32 | 4.7 (2.6 - 5.4) | 407 | 4.4 (2.9 - 5.3) | 213 | 4.7 (3.0 - 5.5) | 0.33 |
| Protein, mg/dL | 24 | 52 (31 - 89) | 361 | 45 (22 - 100) | 195 | 60 (24 - 106) | 0.40 |
| Glucose, mg/dL | 13 | 58 (48 - 85) | 121 | 59 (36 - 96) | 56 | 53 (35 - 100) | 0.98 |
| Values are represented as percentages or median and interquartile range.  Abbreviations:  IQR = Interquartile range, CSF = Cerebral spinal fluid, BP = Blood pressure, CFU = Colony forming units.  *P-values compare across the three groups using the Chi-square test for proportions or the Kruskal-Wallis test for continuous variables.  Cerebral perfusion pressure is calculated as the Mean Arterial Pressure (MAP) minus (Intracranial opening pressure*0.073556127270818), where MAP=(1/3)*systolic BP + (2/3)*diastolic BP | | | | | | | |

| **Supplemental Table 2: Clinical Outcomes and 2-Week Mortality by Baseline Mean Arterial Pressure** | | | | | | | |
| --- | --- | --- | --- | --- | --- | --- | --- |
|  | **Clinical Outcomes by Baseline Systolic Blood Pressure** | | | | | | |
|  | **MAP <70 mmHg** | | **MAP 70 to 100 mmHg** | | **MAP >100 mmHg** |  |  |
| **Number of Participants** | **36** | | | **428** | **222** | **p-value*** |  |
| Incident Seizures (first 7 days), N^1^ | 36 | | | 428 | 222 |  |  |
| N (%) | 2 (6%) | | | 24 (6%) | 24 (11%) | 0.05 |  |
| Day 7 Glasgow Coma Scale score <15, N^1^ | 22 | | | 328 | 149 |  |  |
| N (%) | 9 (41%) | | | 57 (17%) | 39 (26%) | **<0.01** |  |
| Day 14 Glasgow Coma Scale score <15, N^1^ | 18 | | | 218 | 107 |  |  |
| N (%) | 8 (44%) | | | 34 (16%) | 19 (18%) | **<0.01** |  |
|  |  |  | |  |  |  |  |
|  | **2-Week Mortality by Baseline Mean Arterial Pressure** | | | | | | |
|  | **MAP <70 mmHg** |  | | **MAP 70 to 100 mmHg** | **MAP >100 mmHg** |  |  |
| **Number of Participants** | 36 | **p-value*** | | 428 | 222 | **p-value*** |  |
| Number of deaths, N(%) | 13 (36%) |  | | 99 (23%) | 70 (32%) |  |  |
| Unadjusted Hazard Ratio, 95% CI | 1.80 (1.01, 3.20) | **0.047** | | REF | 1.47 (1.08, 1.99) | **0.014** |  |
| Adjusted Hazard Ratio, 95% CI** | 1.33 (0.71, 2.50) | 0.38 | | REF | 1.36 (0.99, 1.86) | 0.06 |  |
| ^1^ N with data.  * P-values for clinical outcomes are from the Chi-square test. P-values for 2-week mortality are from Cox regression models.  ** Models were adjusted for baseline variables of GCS<15, quantitative culture results, and incident seizures(yes/no).  ** Models were adjusted for baseline variables of GCS<15, quantitative culture results, and incident seizures (yes/no).  ** Models were adjusted for baseline variables of GCS<15, quantitative culture results, and seizures (yes/no). | | | | | | | |

| **Supplemental Table 3: Analyses of 2-Week Mortality with a Time-Varying Covariate for Cerebral Perfusion Pressure or Mean Arterial Pressure** | | | | | | |
| --- | --- | --- | --- | --- | --- | --- |
|  | **Deaths, N(%)** | **CPP <70 mmHg or**  **CPP >100 mmHg** | **p-value*** | **Deaths, N(%)** | **MAP <70 mmHg or**  **MAP >100 mmHg** | **p-value*** |
| Unadjusted Hazard Ratio, 95% CI | 166 (16%) | 1.39 (1.02, 1.88) | **0.04** | 182 (11%) | 1.34 (1.0, 1.80) | 0.05 |
| Adjusted Hazard Ratio, 95% CI** | 184 (16%) | 1.27 (0.94, 1.74) | 0.38 | 179 (11%) | 1.24 (0.92, 1.67) | 0.16 |
| * P-values for 2-week mortality are from Cox regression models of CPP and MAP separately.  ** Models were adjusted for baseline variables of GCS<15, quantitative culture results, and incident seizures (yes/no). | | | | | | |

**Figure 1: 2-Week Mortality by Baseline Cerebral Perfusion Pressure**

**
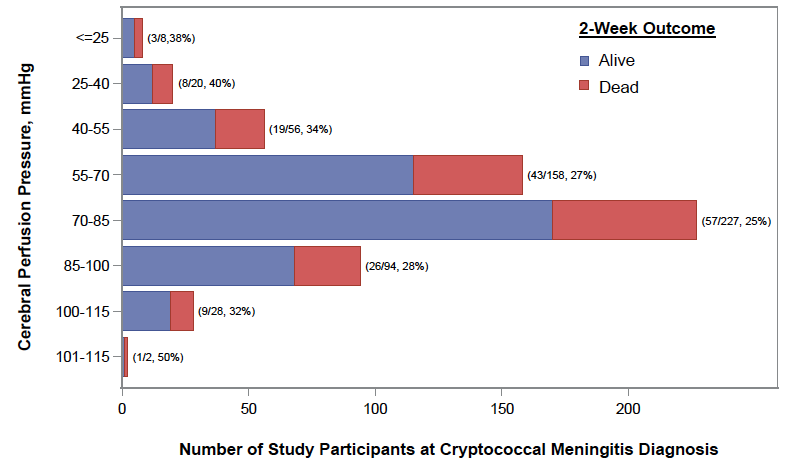
**

**Figure 1:**Barplots representing the number of individuals with cryptococcal meningitis by baseline cerebral perfusion pressure. Percentages represent 2-week mortality.
